# Genome-wide analysis of the diversity and ancestry of Korean dogs

**DOI:** 10.1371/journal.pone.0188676

**Published:** 2017-11-28

**Authors:** Bong Hwan Choi, Hasini I. Wijayananda, Soo Hyun Lee, Doo Ho Lee, Jong Seok Kim, Seok Il Oh, Eung Woo Park, Cheul Koo Lee, Seung Hwan Lee

**Affiliations:** 1 Animal Genome & Bioinformatics, National Institute of Animal Science, RDA, WanJu, Korea; 2 Division of Animal and Dairy Science, Chungnam National University, Daejeon, Korea; 3 Korean Jindo and Domestic Animal Center, Jindo, Korea; 4 Department of Biotechnology, Korea University, Seoul, Korea; Kunming Institute of Zoology, Chinese Academy of Sciences, CHINA

## Abstract

There are various hypotheses on dog domestication based on archeological and genetic studies. Although many studies have been conducted on the origin of dogs, the existing literature about the ancestry, diversity, and population structure of Korean dogs is sparse. Therefore, this study is focused on the origin, diversity and population structure of Korean dogs. The study sample comprised four major categories, including non-dogs (coyotes and wolves), ancient, modern and Korean dogs. Selected samples were genotyped using an Illumina CanineHD array containing 173,662 single nucleotide polymorphisms. The genome-wide data were filtered using quality control parameters in PLINK 1.9. Only autosomal chromosomes were used for further analysis. The negative off-diagonal variance of the genetic relationship matrix analysis depicted, the variability of samples in each population. *F*_*IS*_ (inbreeding rate within a population) values indicated, a low level of inbreeding within populations, and the patterns were in concordance with the results of Nei’s genetic distance analysis. The lowest *F*_*ST*_ (inbreeding rate between populations) values among Korean and Chinese breeds, using a phylogenetic tree, multi-dimensional scaling, and a TreeMix likelihood tree showed Korean breeds are highly related to Chinese breeds. The Korean breeds possessed a unique and large diversity of admixtures compared with other breeds. The highest and lowest effective population sizes were observed in Korean Jindo Black (485) and Korean Donggyeong White (109), respectively. The historical effective population size of all Korean dogs showed declining trend from the past to present. It is important to take immediate action to protect the Korean dog population while conserving their diversity. Furthermore, this study suggests that Korean dogs have unique diversity and are one of the basal lineages of East Asian dogs, originating from China.

## Introduction

Dogs belong to the family Canidae and show high diversity between and among different species. They have diverse feeding habits and advanced social organization. The dog was suggested as the first domesticated animal by archaeological discoveries around the world [1]. Moreover, it is considered as the most distinctive domesticated animal with regard to phenotypic diversity [2]. Behavioral and morphological features, as well as modern genetic evidence, suggest that dogs originated from gray wolves (*Canis lupus*) [1, 2, 3, 4].

There is much interest in determining the ancestry of dogs. Investigating the exact time period for dog domestication will help to clarify wolf and human engagement in the domestication process. It is vital to include Central Asia and other nearby regions, in developing a full picture of early dog history. Since specimens of ancient dogs are unavailable for DNA analysis, some researchers consider village dogs as a reliable sample that resembles ancient dogs [5].

The place of origin of domestic dogs is still inconclusive. There are diverse hypotheses on dog domestication based on various observations. Some literature suggests [6] that dogs have East Asian origin based on osteological features, which are similar to Chinese wolves. In contrast, several archeological studies suggest that domestic dogs originated in Southwest Asia [7].

Genetic information, models of phylogeographic dissimilarity and higher genetic diversity suggest an East Asian origin for domestic dogs [6]. In contrast, Shannon et al. [5] indicated that dogs were domesticated in Central Asia 15,000 years ago through an analysis of autosomal, mitochondrial and Y chromosomal information. Furthermore, Frantz’s study suggested a dual origin for dog domestication based on genomic and archeological evidence [7].

A large number of modern breeds originated from Europe within the past 200 years [8,9]. Among Asian countries, South Korea has a huge interest and demand for dogs. Recently, dogs have been raised for various purposes in South Korea, including as pets, and for, hunting, guarding, and military activities. There are 400 dog breeds worldwide, and among these, more than 150 are bred in South Korea [10]. Accurate determination of relationships among breeds and pedigree registration are vital to sucessful dog breeding.

Korean Jindo White, Korean Poongsan White, Sapsaree, Korean Donggyeong White and Jeju dogs are believed to be native Korean dogs. A microsatellite locus analysis illustrated that Korean native dogs might have ancestry from the northern part of the East Asia [11].

The Korean Jindo dog is a widely known as a hunting and guarding dog. Further, the Korean Jindo White is believed to have been domesticated in the Stone Age. There is a little difference between male and female Jindo dogs (but males are larger than females). The standard height of the Jindo dog is ranges from 45 to 53 cm. They have yellow and white coat colors, and the tail is curled upward [12,13]. The front view of the face is nearly an inverted triangle. The forehead is wide, and the line from the forehead to the muzzle is unbroken. The line from the skullcap to a point between the eyes is longer than the line from the point between eyes to the end of the nose.

The Poongsan breed is considered to be a hunting dog indigenous to North Korea. However, currently the original pedigree of many Poongsan dogs are raised in South Korea [14]. Its height and length range from 55 to 60 cm and 60 to 65 cm, respectively. The Poongsan breed is a relatively large dog. The color of the coat is white and it has a long muzzle. This breed can be differentiated based on a pea-sized bump under its chin, which is a unique characteristic of the Poongsan. [15]

Gyeongju province is a primary area for breeding the Donggyeong dog in Korea. There are nearly 300 animals known to exist. They are friendly to humans, clean and fast. The height and length of female Donggyeong dogs are 45 and 53 cm while those of the male Donggyeong doga are 49 and 57 cm respectively. No tail or a very small tail is one identifying feature of this dog. Generally, they have four coat types: yellow, white, black, and leopard. The Korean Donggyeong has the longest history; therefore, its genetic structure is a valuable resource with great cultural value [16].

There are few scientific studies on the ancestry of Korean dogs. Therefore, this study investigated the genetic diversity, population structure, and origin of Korean dogs, using three Korean breeds (Jindo, Poongsan, and Donggyong). In addition, we compared Korean breeds with worldwide dog populations (ancient and modern breeds) using genome-wide analysis of single nucleotide polymorphisms (SNPs).

## Materials and methods

### Animals and genotype quality control

In total, 2258 animals were used as a sample for this study. To achieve the major objectives of the study, we selected coyote, wolve, and several breeds analyzed in a previous study [10], after reviewing the literature. The Akita (AKT), Chow Chow (CHO), Chinese Shar-Pei (CHS), Lhasa Apso (LHA), Basenji (BSJ), Afghan Hound (AFH), Alaskan Malamute (ALM), Saluki (SAL), Pekingese (PEK), Shiba Inu (SHI), Shi Tzu (SHT), Siberian Husky (SIH), and Tibetan terrier (TIT) dog breeds were categorized as ancient breeds in many publications due to high divergence levels compared to other dogs. It is believed that they originated > 500 years ago [17–18] and are highly associated with the original domestication of dogs [8,19,20]. Furthermore, these breeds can be considered a basal lineage of domestic dogs and live prototypes of ancestral dogs. Therefore, data on these dog breeds were extracted to investigate the relationship between ancient and Korean breeds.

The Border Collie (BDC), Boxer (BOX), Cavalier King Charles Spaniel (CAV), Chinese Crested (CHC),Chihuahua (CHH), Croatian (CRS), English Setter (ENS), English Springer Spaniel (ESS), Great Dane (GRD), Golden Retriever (GRT), German Shepherd (GSD), Maltese (MAL), Miniature Pinscher (MNP), Miniature Schnauzer (MNS), and Newfoundland (NEF) were selected as modern breeds, representing all parts of the world. The sample comprised 1870 modern dog breeds. These breeds emerged during the Victorian era (circa 1830–1900) through controlled breeding practices. Their breeding regime was implemented by humans, and therefore they no longer have a close relationship with wolves [20]. The dog breeds in the sample sizes are indicated in S1 Table.

Korean dogs used in this study included 189 individuals from 6 populations (belonging to three breeds), Korean Poongsan White (KPW), Korean Donggyengi White (KDW), Korean Jindo White (KJW), Korean Jindo Black (KJB), Korean Jindo Black and Tan (KJT), and Korean Jindo Brindle (KJD). Moreover, 7 coyotes and 81 wolves were included in the sample.

Based on memorandum of understanding (MOU) between the research team and the research and breeding center, veterinarians collected blood samples for the research purposes of this study. All blood samples were obtained in an ethical manner, following guidelines for animal health and welfare. Advance approval was acquired from the Institutional Animal Care and Use Committee of the National Institute of Animal Science, of the Rural Development Administration, of South Korea. Genomic DNA from the Korean dogs was isolated from blood samples using standard methods [21]. Samples were genotyped for 173,662 single nucleotide polymorphisms (SNPs) by Illumina CanineHD array. The quality of genome-wide data was maintained by the application of SNP filtering in PLINK 1.9 [22] based on the following quality control parameters: SNPs with low call rates (<90%) or high missing genotypes (>10%) were removed. To reduce bias in the data, the number of minor allele frequencies was limited to 1%. Dog genotypes obtained from other sources [5] were merged into our dataset. Only genotypes from autosomal chromosomes were used for further analysis.

### Diversity, population structure, and phylogenetic analysis

Diversity and population structure analyses were performed using following algorithms: 1) pairwise fixation indices within populations (*F*_IS_) and between populations (*F*_ST_) [23]; 2) heterozygosity and Nei’s standard genetic distance estimation [24]; 3) GRM estimation, 4) multi-dimensional scaling (MDS) analysis; 5) neighbor-joining tree and 6) ancestor’s admixture prediction. The fixation indices, and heterozygosity and Nei’s standard genetic distance analyses were performed using two R packages, hierfstat [25] and StAMPP [26]. GRM was estimated in GCTA v1.25.2 [27]. The four-dimensional pairwise genetic distances matrix was obtained from the calculation of the MDS algorithm in PLINK 1.9 [28] and depicted as a coordinate in R [28]. ADMIXTURE v1.23 [29] was used to detect possible mixtures of ancestral populations by the two to ten adjusted cluster models (K). The neighbor-joining tree was constructed using SNPhylo [30] and depicted in FigTree v1.4.2 [31].

### Migration events, linkage disequilibrium (LD) and demographic estimation

An extended analysis of the relationships among dog populations was performed using TreeMix v1.12 [32]. This approach allows an estimation of possible historical splits and mixtures between populations, termed migration events. A maximum likelihood tree of populations was first produced. We generated a tree model to estimate migration events that may have occurred in the domestication of Korean dogs in relation to both ancient and modern Asian breeds. To account for LD in tree reconstruction, markers were grouped together in windows of 1,000 SNPs. Migration edges that best fit the data were evaluated based on the fraction of the variance defined in the matrix of residuals, in which positive values were preferred. To identify possible introgression traces in dog populations, we generated an *f3* statistical analysis that was introduced [33] using the threepop command line. Three population (A, B, and C) statistical models with significant negative values for both the *f3* statistic and Z-score were selected as a possible event of population B and C introgression in the population A.

Demographic history of the dog population was reflected by the number of estimated recent to past effective population size (*N*_*e*_). *N*_*e*_ was estimated from the LD value following Sved’s equation [34]. Prior to *Ne* calculation, LD was annotated as *r*^*2*^ to measure the correlation of alleles at two loci [35]. We used the default PLINK 1.9 [22] approach and SNeP V1.1 [36] to finalize the estimations of LD and *N*_*e*_. The historical *N*_*e*_ values were plotted using R [28] with the estimated times on the horizontal ordinate.

## Results

### Population structure and diversity

The observed autosomes in the CanineHD array of our genotype data included 140,420 SNPs, as many as in the worldwide dog data obtained from Shannon et al [5]. After the cleaning process, the remaining autosomal SNPs for Korean dogs and other breeds (ancient and modern) were 98.7%, and 93.83%, respectively. The results of population structure analyses are summarized in Table 1.

**Table 1:** Data summary of observed dog populations.

Breed	No. of samples	Observed Heterozygosity	Expected Heterozygosity	*F*_*IS*_^1^	GRM^2^		Adjacent LD(SD)^3^	Recent *Ne*^4^
					Diagonal	Off-diagonal		
**Korean dogs**								
KDW	52	0.41	0.31	-0.24	0.94	-0.20	0.24(0.27)	109
KPW	19	0.41	0.31	-0.24	0.83	-0.05	0.23(0.25)	110
KJW	42	0.4	0.31	-0.22	0.95	-0.02	0.20(0.24)	233
KJB	32	0.4	0.31	-0.22	0.94	-0.03	0.20(0.24)	485
KJD	11	0.4	0.31	-0.23	0.81	-0.08	0.24(0.24)	158
KJT	32	0.4	0.30	-0.22	0.92	-0.03	0.21(0.24)	262
**Ancient dogs**								
AFH	11	0.42	0.30	-0.30	0.72	-0.07	0.29(0.26)	83
AKT	12	0.36	0.27	-0.27	0.69	-0.06	0.28(0.26)	84
ALM	12	0.4	0.30	-0.27	0.76	-0.07	0.28(0.25)	100
BSJ	30	0.4	0.29	-0.24	0.96	-0.03	0.24(0.27)	291
CHO	12	0.37	0.28	-0.25	0.66	-0.13	0.31(0.24)	97
CHS	8	0.4	0.30	-0.23	0.80	-0.11	0.28(0.25)	107
LHA	15	0.44	0.33	-0.25	0.91	-0.06	0.27(0.26)	182
PEK	13	0.42	0.31	-0.27	0.82	-0.07	0.29(0.27)	171
SAL	7	0.43	0.31	-0.28	0.74	-0.12	0.31(0.26)	88
SHI	8	0.39	0.29	-0.25	0.71	-0.10	0.31(0.26)	95
SHT	27	0.42	0.31	-0.26	0.89	-0.03	0.27(0.28)	166
SIH	17	0.4	0.30	-0.24	0.89	-0.06	0.25(0.26)	157
TIT	7	0.44	0.32	-0.30	0.69	-0.12	0.32(0.26)	60

^1 ^Inbreeding coefficients

^2^ Average of the genomic relationship matrix referring to the inbreeding of the animal itself (Diagonal) and referring to the relationship between animals in the population (Off-diagonal)

^3^ Linkage disequilibrium estimated by the *r*^*2*^ method (0–20 Kb marker distance)

^4 ^Effective population size (*Ne*)

Variability of the samples in each population was shown by the negative off-diagonal variances in the GRM analysis. All Korean breeds had relatively high heterozygosity. The observed heterozygosity of the Akita, Shiba Inu and Chow Chow were slightly lower, while other ancient breeds ranged between 0.4 and—0.44.

The inbreeding coefficients (within population *F*_IS_) of Korean breeds were between—0.22 and—0.23 while ancient breeds ranged from -0.23 to -0.3. The *F*_*IS*_ of all dogs observed in this study was negative indicating that the sample used in this study had a low level of inbreeding.

Population differences based on inbreeding coefficient (between populations -*F*_ST_) (Table 2) were used to examine variation within Korean dog populations, as well as their correlation with wolves (gray, Chines, Russian, and Korean) and ancient and modern breeds (Table 2; lower diagonal). Among all selected breeds, Korean Jindo Black had the closest relationship with the Chinese Shar-Pei (*F*_ST_ value 8.079× 10^−2^). The *F*_ST_ values showed that all Korean breeds were closely related to each other and varied between 1.42 ×10^−2^ and 9.338 × 10^−2^. Low *F*_*ST*_ values in Korean breeds suggest low population differentiation. The highest *F*_ST_ value was 35.13 ×10^−2^ between the Tibetan Terrier and Korean wolf, showing that they have the lowest degree of relatedness to each other. With regard to these relationships, Korean breeds were close to Chinese breeds with low *F*_ST_ values, especially Chow Chow and Chinese Shar Pei. Nei’s genetic distance between populations also indicated a close relationship between Chinese and Korean breeds.

**Table 2:** Pairwise *F*_*ST*_ (inbreeding between populations) lower diagonal, and Nei’s genetic distance between populations upper diagonal.

	**AFH**	**AKT**	**ALM**	**BSJ**	**CHO**	**CHS**	**CHW**	**GRW**	**KDW**	**KJB**	**KJD**	**KJT**	**KJW**	**KPW**	**KRW**	**LHA**	**PEK**	**RUW**	**SAL**	**SHI**	**SHT**	**SIH**	**TIT**
**AFH**	0	0.194	0.175	0.159	0.176	0.160	0.212	0.195	0.140	0.133	0.144	0.139	0.134	0.144	0.252	0.134	0.161	0.212	0.130	0.177	0.155	0.154	**0.167**
**AKT**	0.333	0	0.151	0.198	0.123	0.115	0.214	0.178	0.092	0.084	0.093	0.091	0.083	0.103	0.236	0.144	0.170	0.216	0.184	0.124	0.164	0.133	0.184
**ALM**	0.291	0.273	0	0.182	0.138	0.126	0.200	0.176	0.108	0.099	0.111	0.105	0.100	0.114	0.234	0.129	0.154	0.202	0.163	0.140	0.149	0.083	0.164
**BSJ**	0.289	0.344	0.313	0	0.178	0.164	0.209	0.175	0.145	0.138	0.149	0.143	0.139	0.149	0.237	0.142	0.172	0.208	0.147	0.183	0.163	0.160	0.173
**CHO**	0.285	0.225	0.227	0.301	0	0.083	0.202	0.160	0.076	0.063	0.074	0.067	0.063	0.084	0.218	0.128	0.156	0.203	0.166	0.112	0.149	0.117	0.171
**CHS**	0.254	0.203	0.202	0.278	0.113	0	0.183	0.150	0.070	0.058	0.069	0.062	0.058	0.077	0.207	0.112	0.139	0.183	0.150	0.104	0.132	0.106	0.153
**CHW**	0.259	0.276	0.245	0.287	0.233	0.207	0	0.159	0.164	0.161	0.172	0.166	0.162	0.173	0.209	0.171	0.198	0.013	0.200	0.202	0.190	0.185	0.203
**GRW**	0.303	0.297	0.278	0.299	0.244	0.226	0.201	0	0.133	0.124	0.134	0.128	0.124	0.141	0.109	0.163	0.191	0.152	0.186	0.169	0.185	0.157	0.202
**KDW**	0.224	0.163	0.179	0.246	0.116	0.107	0.210	0.211	0	0.028	0.039	0.032	0.028	0.054	0.188	0.094	0.120	0.165	0.130	0.086	0.114	0.088	0.134
**KJB**	0.214	0.149	0.164	0.238	0.087	0.081	0.199	0.196	0.047	0	0.021	0.014	0.010	0.042	0.179	0.088	0.115	0.162	0.124	0.076	0.109	0.079	0.129
**KJD**	0.232	0.168	0.181	0.258	0.102	0.092	0.199	0.207	0.056	0.016	0	0.024	0.020	0.053	0.189	0.100	0.127	0.173	0.135	0.086	0.121	0.090	0.141
**KJT**	0.224	0.163	0.175	0.249	0.098	0.091	0.208	0.206	0.055	0.014	0.024	0	0.013	0.046	0.183	0.093	0.121	0.167	0.130	0.081	0.114	0.084	0.135
**KJW**	0.215	0.148	0.166	0.239	0.090	0.083	0.204	0.198	0.049	0.008	0.016	0.015	0	0.042	0.179	0.089	0.117	0.163	0.126	0.076	0.110	0.080	0.131
**KPW**	0.236	0.186	0.192	0.260	0.130	0.117	0.211	0.223	0.093	0.067	0.078	0.077	0.069	0	0.196	0.101	0.128	0.174	0.134	0.095	0.122	0.094	0.141
**KRW**	0.369	0.375	0.340	0.360	0.322	0.282	0.244	0.150	0.254	0.238	0.259	0.249	0.240	0.272	0	0.220	0.248	0.213	0.244	0.227	0.242	0.215	0.258
**LHA**	0.214	0.237	0.203	0.245	0.184	0.159	0.196	0.241	0.151	0.137	0.146	0.148	0.141	0.159	0.279	0	0.072	0.172	0.123	0.128	0.054	0.110	0.116
**PEK**	0.267	0.290	0.253	0.296	0.243	0.214	0.237	0.289	0.194	0.184	0.200	0.196	0.188	0.206	0.339	0.112	0	0.198	0.151	0.154	0.073	0.138	0.139
**RUW**	0.253	0.269	0.242	0.281	0.230	0.207	0.009	0.192	0.211	0.200	0.199	0.209	0.205	0.210	0.242	0.197	0.234	0	0.200	0.203	0.190	0.186	0.203
**SAL**	0.217	0.304	0.256	0.258	0.247	0.215	0.225	0.272	0.197	0.184	0.197	0.196	0.188	0.203	0.332	0.176	0.232	0.223	0	0.168	0.143	0.143	0.152
**SHI**	0.290	0.232	0.236	0.311	0.181	0.160	0.238	0.263	0.139	0.119	0.134	0.130	0.121	0.155	0.333	0.193	0.247	0.235	0.255	0	0.148	0.123	0.167
**SHT**	0.257	0.278	0.247	0.284	0.235	0.210	0.241	0.284	0.191	0.183	0.196	0.193	0.186	0.204	0.328	0.089	0.130	0.239	0.225	0.239	0	0.131	0.132
**SIH**	0.255	0.238	0.146	0.280	0.187	0.167	0.227	0.249	0.150	0.133	0.146	0.144	0.136	0.159	0.300	0.175	0.226	0.226	0.222	0.203	0.220	0	0.147
**TIT**	0.269	0.305	0.259	0.293	0.256	0.221	0.229	0.291	0.203	0.192	0.207	0.203	0.196	0.213	0.351	0.167	0.218	0.227	0.224	0.258	0.211	0.228	0

AFH: Afghan Hound, AKT: Akita, ALM: Alaskan Malamute, BSJ: Basenji, CHO: Chow Chow, CHS: Chinese Shar Pei, CHW: Chinese wolf, GRW: Gray wolf, KDW: Korean Donggyengi, KJB: Korean Jindo Black, KJD: Korean Jindo Brindle, KJT: Korean Jindo Black and Tan, KJW: Korean Jindo White, KPW: Korean Poongsan White, KRW: Korean wolf, LHA: Lhasa Apso, PEK:Pekingese, RUW: Russian wolf, SAL: Saluki, SHI: Shiba Inu, SHT: Shih Tzu, SIH: Siberian Husky, TIT:Tibetan Terrier

**Fig 1:**
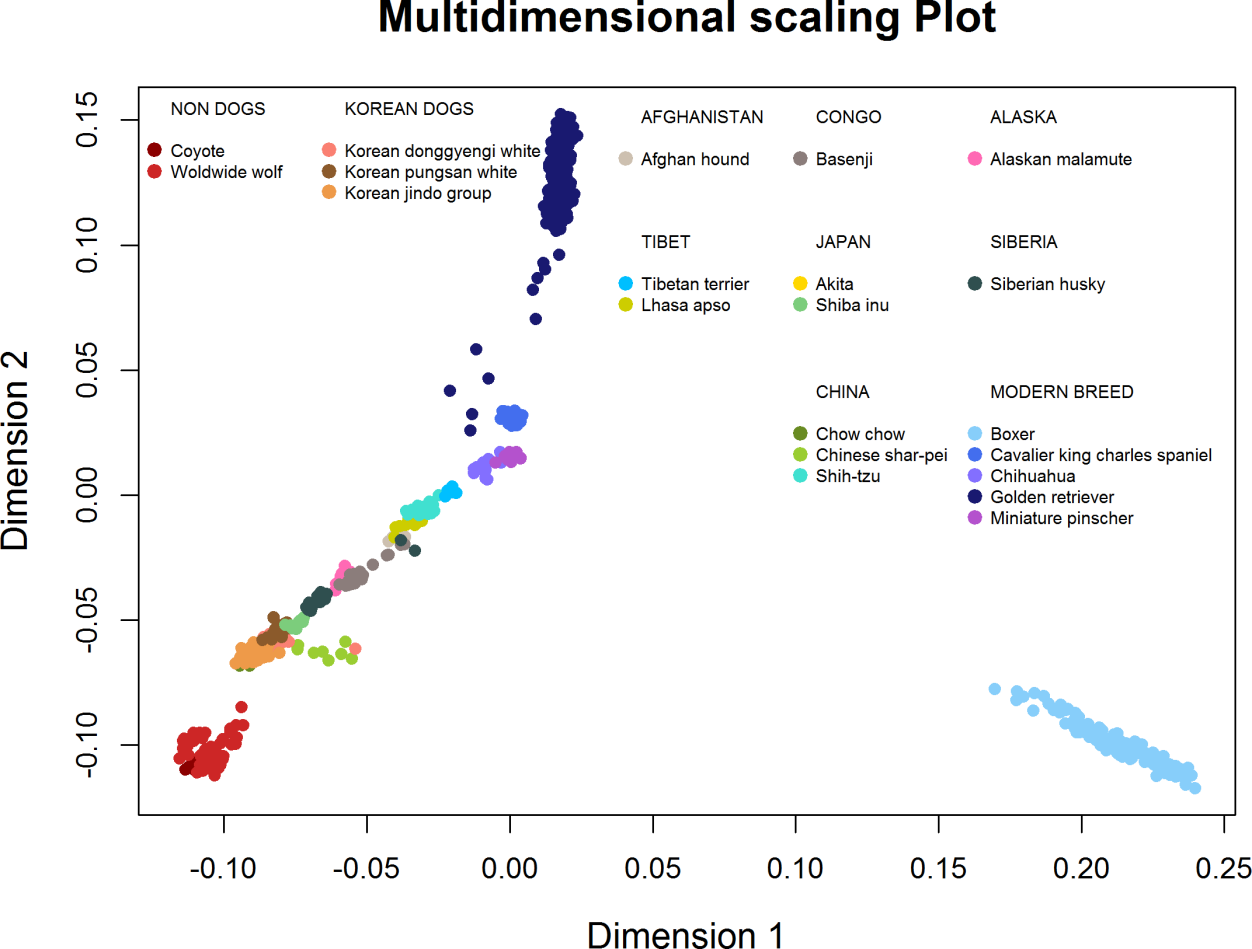
Multi-dimensional scaling (MDS) plot of Korean dogs compared to ancient and selected modern breeds. Points were separated using colors to differentiate each dog breed.

The MDS results are depicted in Fig 1. The plot was constructed using coyotes, worldwide wolves, Korean dogs, and dogs from other parts of the world. MDS analysis allows visualization of the genetic distance of each breed within a selected sample. Various colors were used to differentiate breeds. The group containing wolves was placed in the left corner. All Korean breeds were situated near the non-dog group and were tightly clustered with each other. Chinese Shar-Pei, Chow Chow, and Shiba Inu clustered with the Korean breeds. European breeds such as Cavalier King Charles Spaniel, Chihuahua, Golden Retriever, and Miniature Pinscher were located further away from the wolves and Korean breeds. In particular, the Boxer was located furthest away from all other breeds at a great distance.

### Population ancestries and migration events

**Fig 2:**
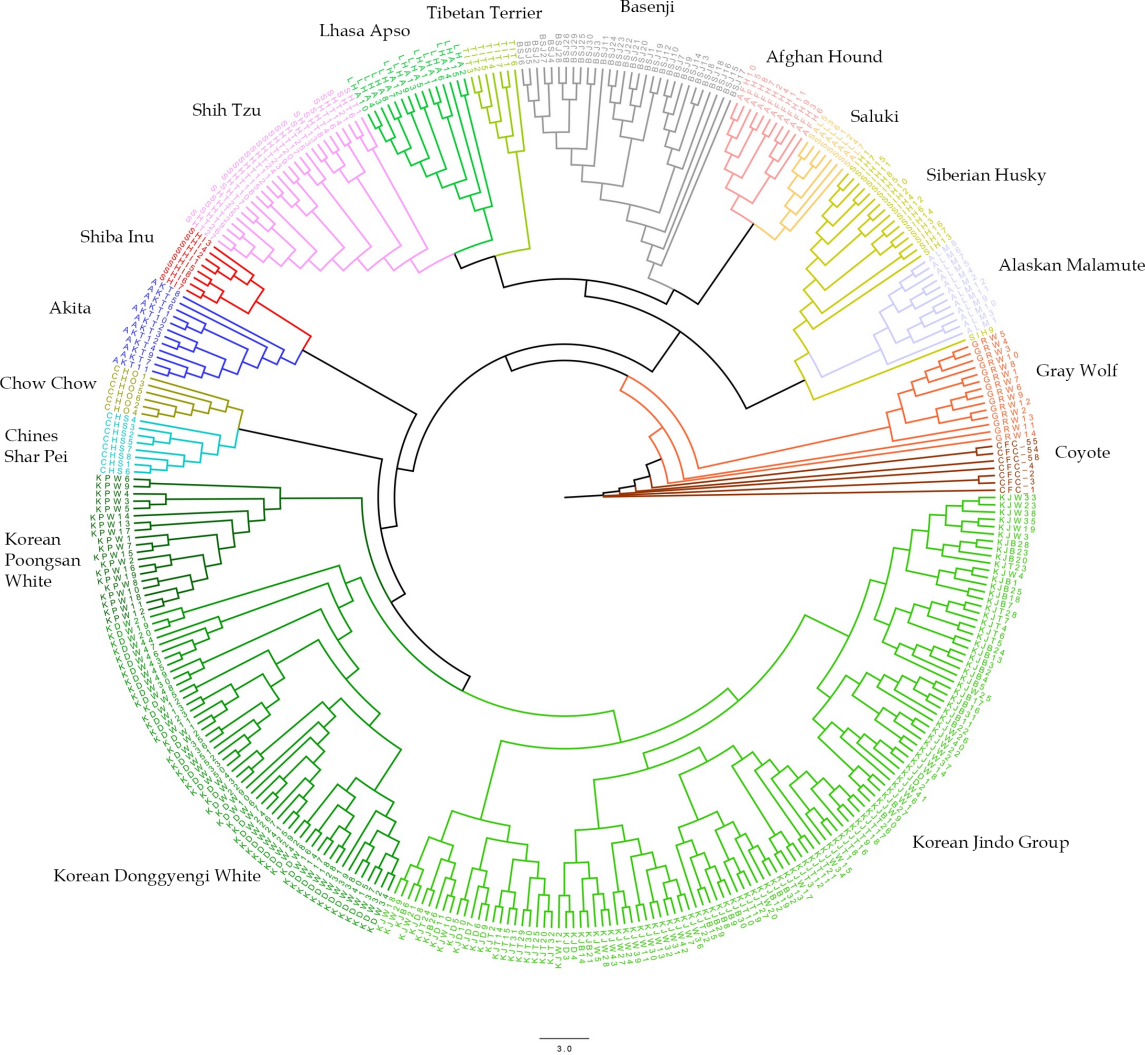
Neighbor-joining tree of Korean dogs compared to coyote, gray wolf, and ancient dogs. Neighbor-joining tree including coyote (CFC), gray wolf (GRW) Donggyeong white (KDW), Poongsan White (KPW), Jindo White (KJW), JindoBblack (KJB), Jindo Brindle (KJD), Korean Jindo Black and Tan (KJT), Afghan Hound (AFH), Akita (AKT), Alaskan Malamute (ALM), Basenji (BSJ), Chow Chow (CHO), Chinese Shar Pei (CHS), Lhasa Apso (LHA), Saluki (SAL), Shiba Inu (SHI), Shi Tzu (SHT), Siberian Husky (SIH)and Tibetan Terrier (TIT). The phylogeny was rooted with the coyote. Colors were used to differentiate among dog breeds, with Korean breeds indicated by different shades of green color.

**Fig 3:**
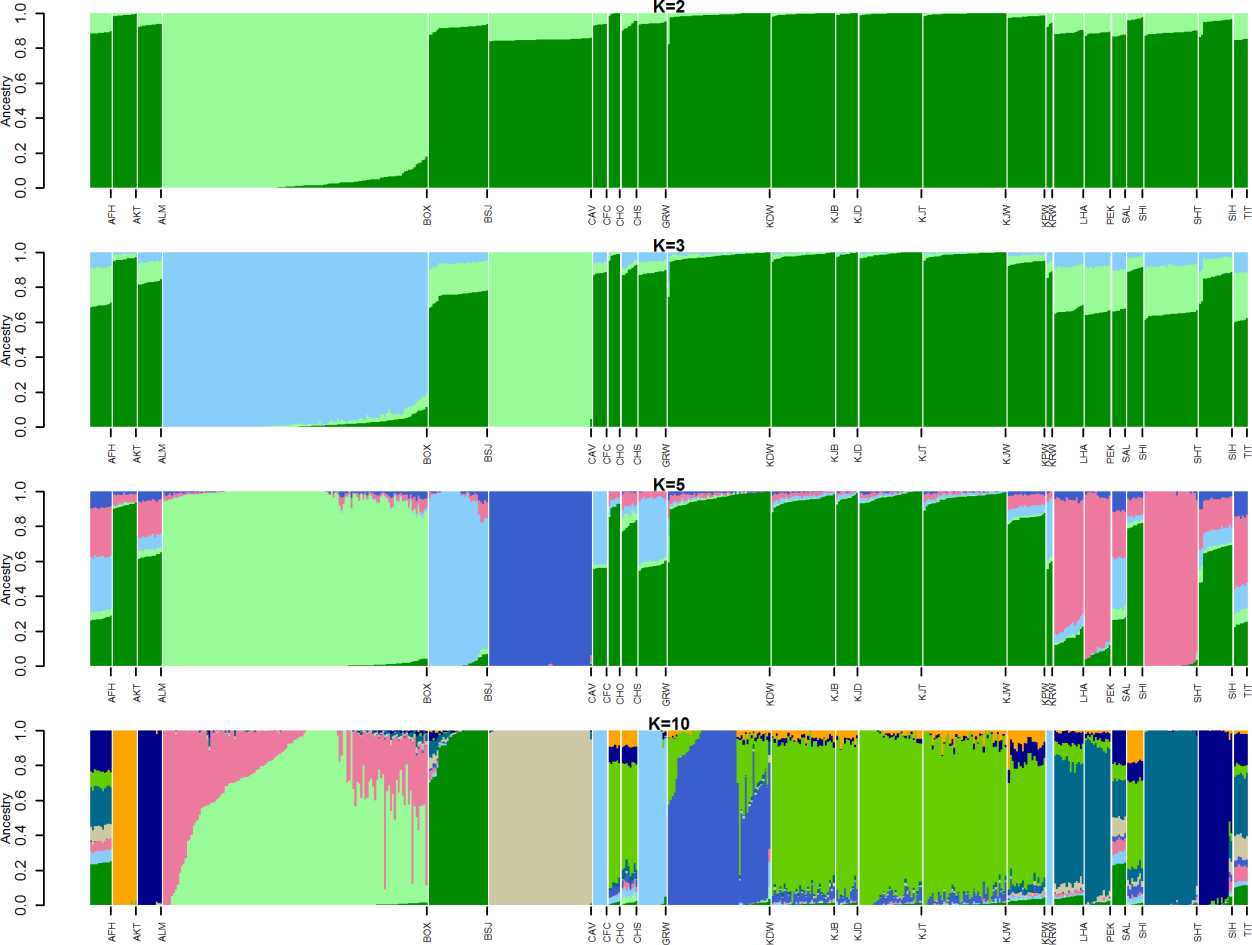
Ancestry model for Korean breeds including ancient and selected modern breeds. Each vertical line represents one individual. Admixture results include coyote (CFC), Korean wolf (KRW), Donggyeong White (KDW), Poongsan White (KPW), Jindo White (KJW), Jindo Black (KJB), Jindo Brindle (KJD), Korean Jindo Black and Tan(KJT), Afghan Hound (AFH), Akita (AKT), Alaskan Malamute (ALM), Basenji (BSJ), Chow Chow (CHO), Chinese Shar Pei (CHS), Lhasa Apso (LHA), Saluki (SAL), Shiba Inu (SHI), Shi Tzu (SHT), Siberian Husky (SIH), Tibetan Terrier (TIT), Boxer (BOX), and Cavalier King Charles Spaniel. Phylogeny was rooted in the coyote. K refers to the number of estimated ancestors, as differentiated by colors. The model started at K = 2.

**Fig 4:**
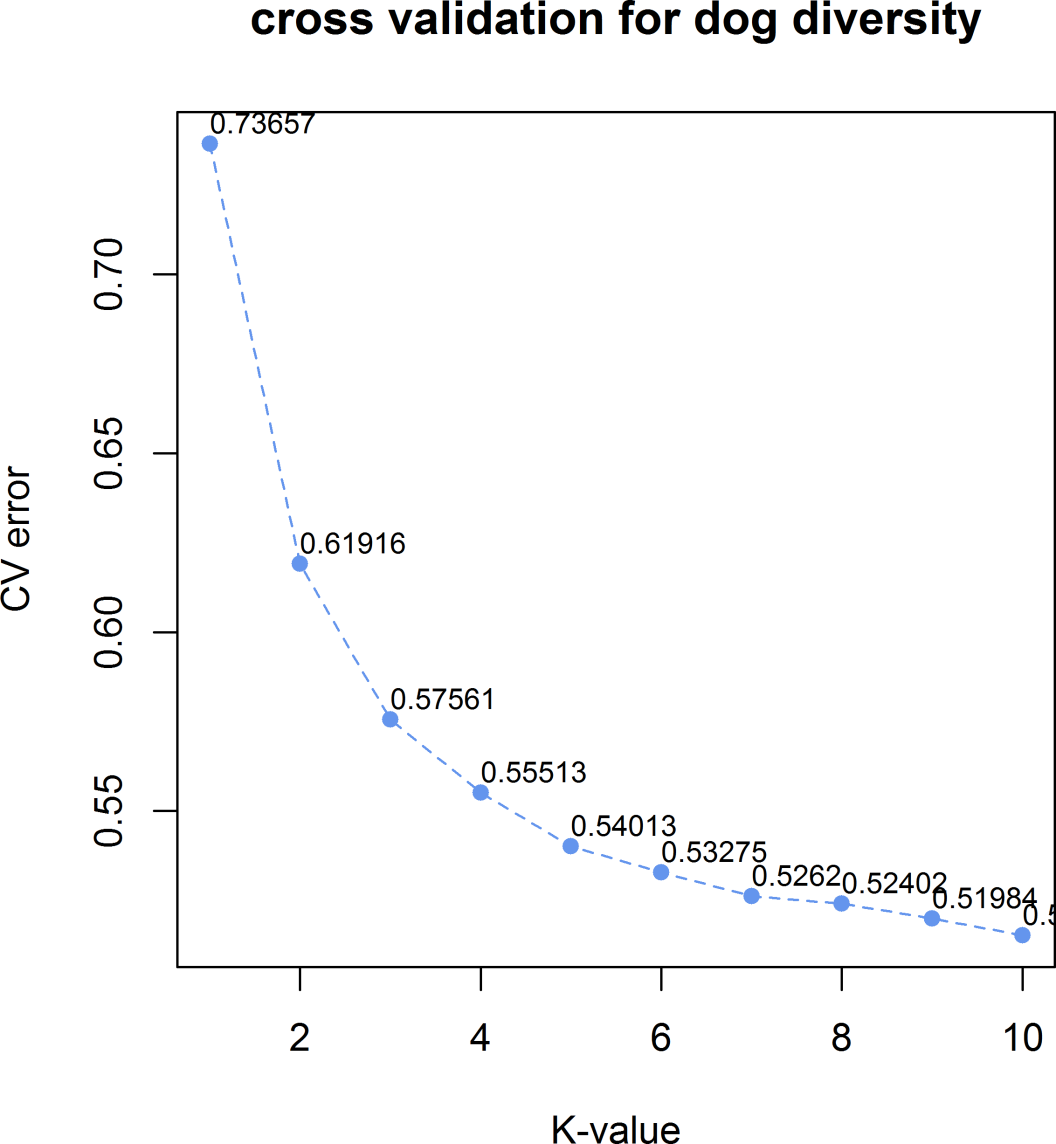
Cross-validation plot of admixture analysis. Admixture with cross-validation for K values 2,3,5, and 10.

**Fig 5:**
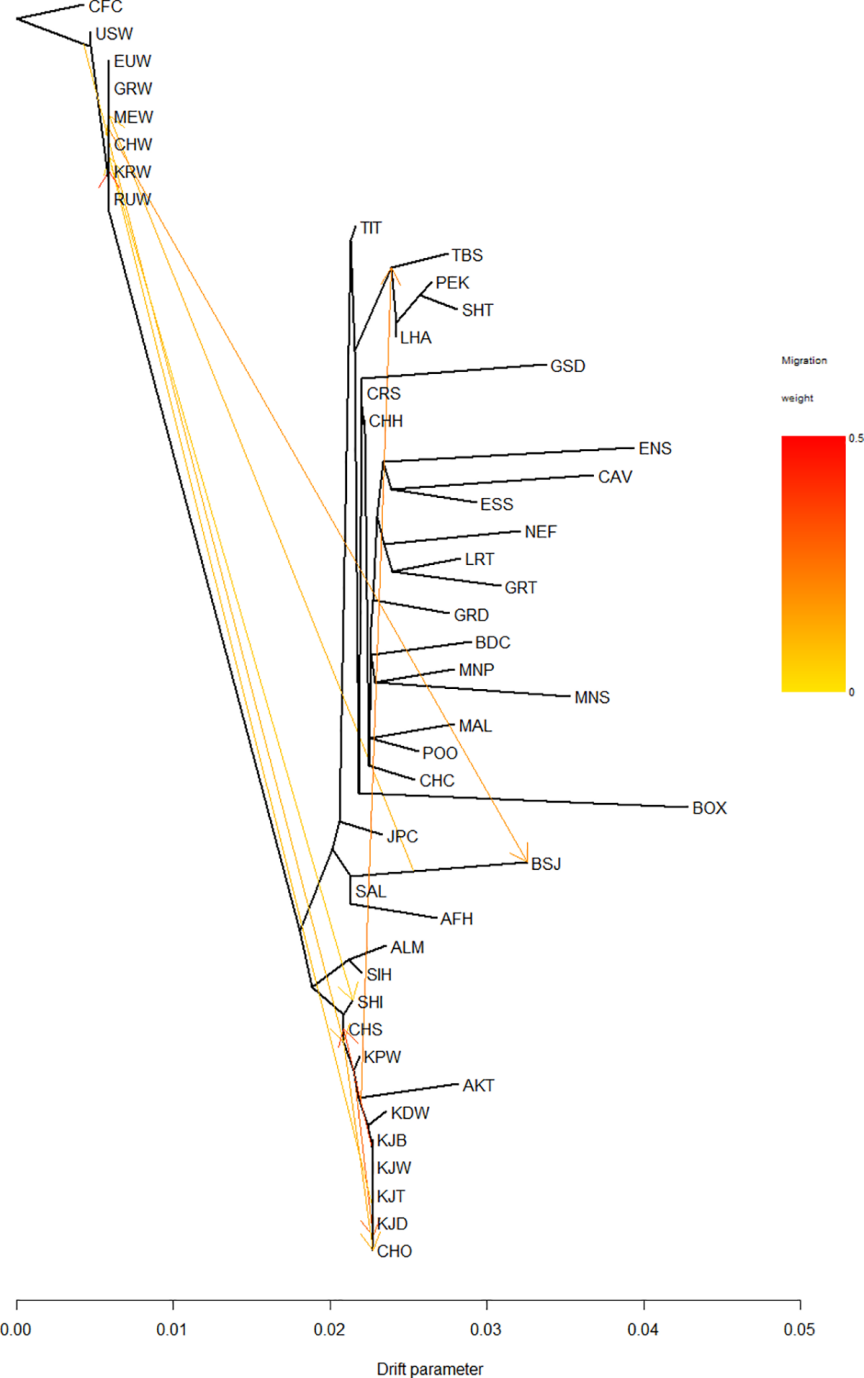
Maximum likelihood tree with migration events. Coyote (CFC) was selected as the root.Gray wolf (GRW), Korean wolf (KRW), Chinese wolf (CHW), European wolf (EUW),Mediterranean wolf (MEW), Russian wolf (RUW), US wolf (USW), Donggyeong White (KDW), Poongsan white (KPW), Jindo White (KJW), Jindo Black (KJB), Jindo Brindle (KJD), Korean Jindo Black and Tan (KJT), Afghan Hound (AFH), Akita (AKT), Alaskan Malamute (ALM), Basenji (BSJ), Chow Chow (CHO), Chinese Shar Pei (CHS), Lhasa Apso (LHA), Saluki (SAL), Shiba Inu (SHI), Shi Tzu (SHT), Siberian Husky (SIH),Tibetan Terrier (TIT), Border Collie (BDC), Boxer (BOX), Cavalier King Charles Spaniel (CAV), Chinese Crested (CHC), Chihuahua (CHH),Croatian (CRS), English Setter (ENS), English Springer Spaniel (ESS), Great Dane (GRD), Golden Retriever (GRT), German Shepherd (GSD), Japanese Chin (JPC), Labrador Retriever (LRT), Maltese (MAL), Miniature Pinscher (MNP), Miniature Schnauzer (MNS), Newfoundland (NEF) and Poodle (POO). Migration boundaries are denoted with arrows in the direction from the migrant’s origin to the recipient breed and heat colored according to the mixture percentage.

Neighbor-joining tree (Fig 2), admixture (Fig 3 and Fig 4), and TreeMix (Fig 5 and S1 Fig) analyses were used to determine viable Korean dog ancestries. The neighbor-joining tree was constructed using the coyote, gray wolf, and ancient and Korean dogs. Coyote was selected as the root of the tree. The tree had two main branches. Siberian Husky and Alaskan Malamute (morphologically wolf-like dogs) formed another one sub clade next to the root. Afghan Hound, Basenji, Tibetan Terriers, Lhasa Apso, and Shi Tzu formed another branch, similar to a previous study [8], Shih Tzu and Lhasa Apso, which have similar appearances, were grouped in a single clade. The next branch was situated further away from the previous breeds and consisted of the Shiba Inu, Akita, Chow Chow, Chines Shar Pei and all Korean breeds. All Korean Poongsan White, Korean Donggyeong white, Korean Jindo Brindle, Korean Jindo Black, Korean Jindo White and Korean Jindo Black and Tan were found in a single clade.

The results of the admixture analysis clearly show the genetic structure of Korean dogs in an ancestry-based model (Fig 3). We conducted admixture analysis with K = 2, K = 3, K = 5 and K = 10 and revealed that the lowest error after cross-validation was obtained with K = 10 (cross-validation error = 0.5153, Fig 4). K = 2, K = 3, K = 5, and K = 10 were selected to improve visualization of the ancestry model while displaying the relationship among Korean, ancient and modern breeds.

The admixture results of K = 10 clearly showed the diversity and admixture of Korean breeds compared with other breeds. Although Korean dogs were admixed with both the ancient and wolf categories, they showed a distinctive admixture compared with all dogs in the sample. Korean Donggyeong White had a distinct genetic makeup from Jindo and Poongsan. Admixture analysis also showed a strong relationship among Chow Chow, Shar-Pei and Korean breeds. Akita, Alaskan Malamute, Basenji, Shi Tzu, Siberian Husky and Cavalier King Charles Spaniel showed very low levels of admixture. Korean breeds showed admixture events with some Japanese breeds, such as Akita and Shiba Inu. Close relationships among coyote, gray wolf, and Korean wolf were visualized in this analysis.

Several migration events of Korean dogs were revealed using non-dogs, and ancient and modern dogs in the maximum likelihood tree (Fig 5). Migration edges that best fit the data were selected if they had positive values as seen in a plot of residuals (S1 Fig) with basal colors. The coyote was set as the root of the ancestry model. The tree showed that all Korean breeds were clustered in one branch with some ancient Chinese and Japanese dogs. The modern breeds clearly clustered together away from wolves while the Boxer exhibited the highest genetic drift in the sample.

Several migration events could be observed in the TreeMix results. A few important migrations were observed from Korean Jindo Black to the Chinese Shar Pei, Akita to Tibetan spaniel and wolf clade to Basenji with a high migration weight. Observation of the residuals from the fit of the model to the data (S1 Fig) revealed that a number of populations do not adhere to a strict tree model.

The *f3* statistics were generated to trace the possible ancestry mixtures in Korean dogs using a sample that included ancient breeds, and the gray wolf. A concise table of the most significant *f3* statistics (standardized to a Z score <—2) is shown in Table 3. Coyote and European wolf introgression on Russian wolf were significant.

**Table 3:** The most significant *f3* statistics shown the possible ancestor mixture of Korean, ancient dog populations and outgroup.

Population A	Population B	Population C	*F3* statistics	Standard Error	Z-Score
Gray wolf	Coyote	Russian wolf	-0.0004	0.0004	-1.1721
Gray wolf	European wolf	Coyote	-0.0013	0.0003	-4.4004
Korean Jindo Black	German Shepherd	Korean Jindo Black and Tan	-0.0002	0.0004	-0.5669
Russian wolf	Chinese wolf	European wolf	-0.0003	0.0002	-1.796
**Russian wolf**	**European wolf**	**Coyote**	**-0.0005**	**0.0002**	**-2.2198**
Russian wolf	Korean wolf	European wolf	-0.0004	0.0002	-1.5203

Most significant *f3* results are indicated in bold.

### Demographic trends

**Fig 6:**
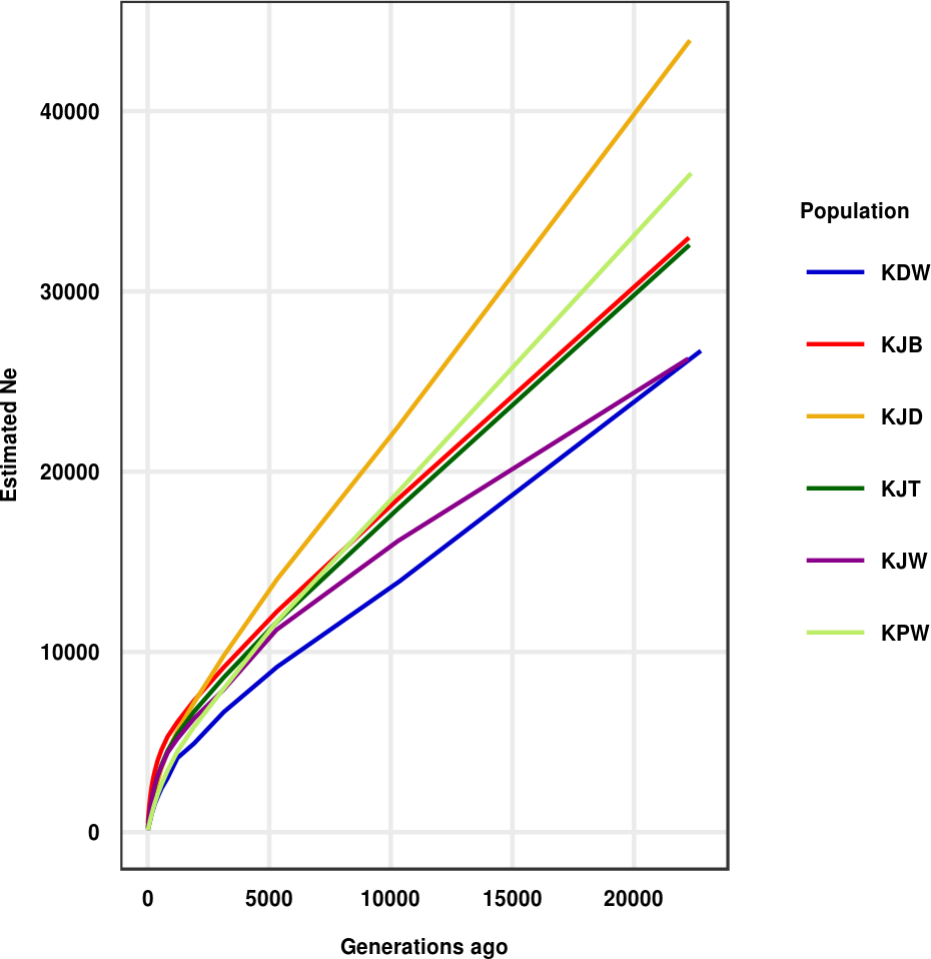
Historical trends in the effective population size of Korean dogs. Trends of the effective population size range from ~12 to 25,000 generations. Lines are colored based on breeds.

The historical effective population size values were estimated based on the LD value across the genome and were used as a representation of demographic changes in the dog population. The adjacent LD (0–20 Kb marker distance) and recent *Ne* values of the observed dog breeds are summarized in Table 1 and averaged in Table 4 based on genetic distance ranges. *Ne* over ~20,000 generations is shown in Fig 6. All Korean dogs have low adjacent LD values than ancient breeds (Table 2). The highest effective population size (*Ne*) for Korean dogs was recorded twelve generations ago for the Korean Jindo Black (485), followed by these populations, in decreasing order: Jindo Black and Tan (262), Korean Jindo White (233), Korean Jindo Brindle (158), Poongsan White (110), Korean Donggyeong White (109).

**Table 4:** Historical effective population size (*Ne*).

**KDW**																					
GenAgo	12	14	16	19	23	27	32	38	46	56	69	85	106	135	173	226	301	410	571	818	1222
Ne	109	124	138	158	182	207	240	280	326	389	465	553	656	843	1045	1262	1597	1950	2437	3005	4113
r2	0.0739	0.0748	0.0770	0.0779	0.0791	0.0812	0.0825	0.0838	0.0859	0.0869	0.0884	0.0909	0.0944	0.0934	0.0959	0.1016	0.1055	0.1144	0.1241	0.1391	0.1486
**KPW**																					
GenAgo	12	14	16	19	23	27	32	38	46	56	69	85	106	135	173	226	301	410	571	819	1221
Ne	110	124	139	160	183	209	240	281	329	387	464	560	680	838	1037	1302	1651	2084	2655	3420	4474
r2	0.1069	0.1080	0.1099	0.1109	0.1121	0.1138	0.1159	0.1171	0.1186	0.1206	0.1218	0.1234	0.1254	0.1273	0.1299	0.1328	0.1363	0.1422	0.1498	0.1597	0.1728
**KJW**																					
GenAgo	12	14	16	19	23	27	32	38	46	56	69	85	106	135	173	226	301	409	571	820	1222
Ne	233	263	300	342	388	443	511	596	691	802	952	1100	1324	1564	1844	2234	2650	3113	3661	4418	5183
r2	0.0501	0.0507	0.0512	0.0519	0.0528	0.0537	0.0545	0.0552	0.0564	0.0578	0.0588	0.0611	0.0625	0.0652	0.0688	0.0721	0.0776	0.0856	0.0963	0.1088	0.1293
**KJB**																					
GenAgo	12	14	16	19	23	27	32	38	46	56	69	85	106	135	173	226	301	410	571	820	1222
Ne	485	542	591	648	727	807	900	1014	1144	1284	1470	1686	1936	2236	2586	2982	3423	3977	4578	5303	6110
r2	0.0441	0.0445	0.0454	0.0463	0.0469	0.0479	0.0489	0.0499	0.0512	0.0527	0.0542	0.0559	0.0581	0.0606	0.0637	0.0679	0.0734	0.0803	0.0900	0.1030	0.1222
**KJD**																					
GenAgo	12	14	16	19	23	27	32	38	46	56	69	85	106	135	173	226	301	409	571	819	1221
Ne	158	180	205	234	267	309	361	419	492	575	690	818	977	1192	1458	1809	2225	2815	3548	4443	5609
r2	0.1294	0.1299	0.1306	0.1314	0.1324	0.1332	0.1338	0.1350	0.1360	0.1377	0.1385	0.1405	0.1427	0.1445	0.1471	0.1499	0.1544	0.1588	0.1655	0.1754	0.1891
**KJT**																					
GenAgo	12	14	16	19	23	27	32	38	46	56	69	85	106	135	173	226	301	410	571	820	1222
Ne	262	293	328	366	415	466	536	611	696	798	931	1087	1280	1517	1802	2156	2579	3110	3724	4511	5436
r2	0.0547	0.0555	0.0564	0.0575	0.0584	0.0597	0.0606	0.0619	0.0635	0.0654	0.0670	0.0690	0.0713	0.0739	0.0772	0.0812	0.0865	0.0931	0.1025	0.1146	0.1323
**AFH**																					
GenAgo	12	14	16	19	23	27	32	38	46	56	69	85	106	135	173	226	301	409	571	819	1221
Ne	83	89	97	105	115	128	142	160	183	214	251	297	358	437	547	687	890	1177	1552	2111	2970
r2	0.1614	0.1666	0.1711	0.1769	0.1821	0.1874	0.1934	0.1988	0.2034	0.2073	0.2119	0.2165	0.2206	0.2249	0.2278	0.2326	0.2357	0.2392	0.2464	0.2535	0.2614
**AKT**																					
GenAgo	12	14	16	19	23	27	32	38	46	56	69	85	106	135	173	226	301	410	571	819	1221
Ne	84	88	95	104	112	124	140	157	180	212	248	295	358	441	553	694	891	1187	1582	2171	3032
r2	0.1533	0.1594	0.1652	0.1703	0.1766	0.1828	0.1872	0.1928	0.1977	0.2009	0.2056	0.2095	0.2129	0.2161	0.2189	0.2237	0.2280	0.2307	0.2363	0.2422	0.2510
**CHO**																					
GenAgo	12	14	16	19	23	27	32	38	46	56	69	85	106	135	173	226	301	410	571	819	1221
Ne	97	108	122	136	153	175	200	229	270	313	370	453	546	676	844	1064	1377	1817	2376	3258	4561
r2	0.2277	0.2297	0.2317	0.2343	0.2369	0.2389	0.2414	0.2444	0.2461	0.2493	0.2520	0.2528	0.2556	0.2575	0.2599	0.2630	0.2653	0.2681	0.2740	0.2784	0.2848
**CHS**																					
GenAgo	12	14	16	19	23	27	32	38	46	56	69	85	106	135	173	226	301	410	571	819	1222
Ne	107	118	132	147	165	189	217	250	288	344	412	498	607	760	947	1207	1525	1987	2608	3431	4735
r2	0.1808	0.1833	0.1854	0.1880	0.1907	0.1924	0.1944	0.1968	0.1998	0.2007	0.2022	0.2040	0.2058	0.2066	0.2090	0.2109	0.2150	0.2186	0.2238	0.2317	0.2393
**LHA**																					
GenAgo	12	14	16	19	23	27	32	38	46	56	69	85	106	135	173	226	301	409	571	820	1222
Ne	182	194	207	223	244	267	290	325	362	414	474	547	640	758	925	1132	1403	1764	2232	2874	3738
r2	0.1001	0.1029	0.1060	0.1091	0.1119	0.1153	0.1195	0.1228	0.1270	0.1304	0.1345	0.1390	0.1436	0.1485	0.1524	0.1577	0.1637	0.1708	0.1801	0.1915	0.2072
**PEK**																					
GenAgo	12	14	16	19	23	27	32	38	46	56	69	85	106	135	173	226	301	410	571	819	1222
Ne	171	177	186	197	203	216	230	251	274	304	343	388	450	527	641	780	977	1262	1648	2204	3018
r2	0.1124	0.1164	0.1203	0.1247	0.1308	0.1362	0.1426	0.1485	0.1550	0.1618	0.1683	0.1759	0.1828	0.1905	0.1961	0.2038	0.2105	0.2166	0.2247	0.2337	0.2453
**SAL**																					
GenAgo	12	14	16	19	23	27	32	38	46	56	69	85	106	135	173	226	301	410	571	819	1220
Ne	88	98	108	120	133	150	172	197	229	269	323	389	472	573	713	913	1151	1493	1990	2691	3739
r2	0.2098	0.2122	0.2155	0.2188	0.2227	0.2265	0.2287	0.2321	0.2350	0.2377	0.2395	0.2418	0.2444	0.2483	0.2513	0.2533	0.2586	0.2636	0.2684	0.2750	0.2832
**SHI**																					
GenAgo	12	14	16	19	23	27	32	38	46	56	69	85	106	135	173	226	301	409	571	819	1221
Ne	95	102	111	122	134	150	169	193	225	262	307	366	441	546	685	861	1113	1453	1958	2659	3721
r2	0.1873	0.1920	0.1956	0.1999	0.2045	0.2083	0.2122	0.2159	0.2188	0.2220	0.2260	0.2293	0.2329	0.2350	0.2374	0.2413	0.2443	0.2486	0.2524	0.2585	0.2660
**SHT**																					
GenAgo	12	14	16	19	23	27	32	38	46	56	69	85	106	135	173	226	301	410	571	820	1222
Ne	166	169	175	184	194	209	225	244	271	302	344	391	455	540	647	790	985	1249	1603	2119	2829
r2	0.0736	0.0784	0.0832	0.0879	0.0934	0.0983	0.1041	0.1103	0.1161	0.1224	0.1282	0.1353	0.1420	0.1483	0.1553	0.1625	0.1698	0.1781	0.1883	0.1991	0.2147
**SIH**																					
GenAgo	12	14	16	19	23	27	32	38	46	56	69	85	106	135	173	226	301	410	571	819	1221
Ne	157	167	180	194	212	231	257	288	324	370	427	501	595	717	877	1084	1347	1726	2210	2856	3791
r2	0.0975	0.1007	0.1038	0.1073	0.1106	0.1145	0.1180	0.1218	0.1258	0.1296	0.1337	0.1373	0.1411	0.1449	0.1489	0.1535	0.1595	0.1650	0.1733	0.1843	0.1976
**TIT**																					
GenAgo	12	14	16	19	23	27	32	38	46	56	69	85	106	135	173	226	301	409	571	819	1221
Ne	60	66	74	83	95	109	125	146	169	202	243	295	367	453	571	724	950	1262	1686	2324	3311
r2	0.2378	0.2417	0.2451	0.2487	0.2519	0.2541	0.2572	0.2595	0.2637	0.2653	0.2671	0.2691	0.2698	0.2725	0.2749	0.2783	0.2798	0.2826	0.2878	0.2928	0.2986

KDW: Korean Donggyongi White, KPW: Korean Poongsan White, KJW:Korean Jindo white, KJB: Korean Jindo Black KJD: Korean Jindo Brindle KJT: Korean Jindo Black, AFH: Afghan Hound, AKT: Akita; CHO: Chow Chow, CHS: Chines Shar Pei, LHA: Lhasa Apso, PEK: Pekingese, SAL: Saluki, SHI: Shiba Inu, SHT: Shi Tzu; SIH: Siberian Husky; TIT: Tibetan Terrier

The effective population size (*Ne*) of all Korean dogs exhibited a declining pattern from the past to recent times (Fig 6). This has caused a decrease in the inbreeding rate from the past to present in Korean dog breeds. The *Ne* trend for Korean Donggyeong White and Korean Jindo White can be traced back to 239,233 (while other breeds can be traced back to more than ~1,000,000 years ago Table 4).

## Discussion

This study was based on genome-wide SNP data to reveal information on diversity, population structure, ancestry, migration events, and demographic trends compared with ancient, and modern breeds and their ancestors (wolves and coyotes). Dogs originated from the gray wolf, and various studies have presented diverse hypotheses for dog domestication [37, 38]. Although a considerable number of studies used different methods, they had various drawbacks and information on the ancestry of Korean dogs is rare. Data based on genome-wide SNPs are appropriate for these types of studies and some previous studies have used this kind of data. However, most of these studies have lacked samples from Northeast Asia, especially from Korea. Therefore, this study mainly focused on the diversity and ancestry of Korean dogs and revealed interesting information about these dogs.

Ascertainment bias is the systematic variation of population genetic statistics from theoretical expectations. It occurs due to sampling a non-random set of individuals, small sample sizes, or biased SNP discovery protocols [39]. Moreover, small sample size tends to bias towards common SNPs in the allele frequency distribution [40]. This error always occurs, unless sequencing is performed on the whole genome of every individual. High coverage sequencing data, analysis of a large number of SNPs [41,42], raw data modification, and incorporating ascertainment bias into the theoretical models of population genetics can minimize this error [39]. The ascertainment bias in our analysis was minimized by using a considerable sample size, a large SNP genotype dataset and through sample size correction protocols. Therefore, the present study provides precise results on Korean dog ancestry.

The data used in this study were grouped into four different categories to improve the clarity of the analysis. GRM analysis was performed for all Korean breeds and ancient dogs. The heterozygosity in Korean dogs was high (around 0.4), while the inbreeding coefficient within populations indicates that all Korean breeds in this study had a low level of inbreeding. Previously, it was revealed that Korean Donggyeong White, Korean Jindo White, and Korean Poongsan White had heterozygosity values of 0.77,0.70, and 0.74, respectively [43]. The sample of this study has a low level of heterozygosity compared to that study. Lee et al. [44] showed an average inbreeding coefficient within populations of Korean breeds of 0.028. The inbreeding coefficient is comparatively higher than this study. Ancient history and recent factors such as breeding programs introduced during the past few hundred years can lead to changes in the genetic diversity of individuals. Nevertheless, the variation may be due to the differentiation between samples and different methodologies used in the studies [45].

*F*_ST_ values were used to investigate genetic diversity between populations. Korean breeds showed more similar allele frequencies with some Chinese breeds (Chow Chow and Chinese Shar Pei.) than others in the sample. The MDS, TreeMix and admixture results also indicated close relationships between Korean and Chinese breeds. MDS analysis showed that Korean breeds are closely related to wolves. The modern breeds show a distinct genetic background from their dog ancestors. It was previously found that Southeast Asian dogs were closely related to wolves, especially Chow Chow, Akita and Chinese Shar Pei. Further, they are considered a foundation lineage connecting to the gray wolf [6,45,46, 47]. Fan et al. [48] found that the Boxer genome does not follow any wolf population, which agrees with our results.

Some publications clearly established that gray wolves (*C*. *lupus*) are distributed throughout China in both ancient and modern times [49]. According to Wang et al., [45] wolves from the southern part of East Asia have a significant genetic relationship with domestic dogs. All of these studies shed light on East Asian dog domestication. The results of our study are in significant agreement with these previous studies. Because there is little literature showing the close relationship among Chinese wolves, Korean wolves, and dogs, our observations represent a reliable source of information for future studies.

The phylogenetic tree, MDS, admixture analysis, and TreeMix results provide evidence showing that Korean dogs have a close relationship with Japanese breeds. A previous study also revealed that Korean dogs were brought to Japan many years ago [50].

The admixture analysis revealed that Korean breeds are uniquely diverse compared with all other breeds, although they were admixed with both wolf and ancient dog breeds. Korean Donggyeong White showed a different genetic makeup from when compared to other Korean breeds. Nevertheless, most of the migration events could not be identified from the F statistics due to the difficulty in identifying admixtures due to the large amount of genetic drift since the admixture event [51].

Effective population size is the main factor in population genetics and conservation [51] because it strongly associated with inbreeding, fitness and loss of genetic variation through random genetic drift [52, 53]. Therefore, it is considered as an important criterion for determining the endengerment of a population [54,55].

The historical effective population size suggests that all Korean breeds exhibit decreasing effective population sizes over long time scales. The results of this analysis are agree with a previous study of effective population size in the Sapsaree breed [56]. The smallest effective population size were observed in the Korean Poongsan White and Korean Donggyeong White breeds, while the largest effective population size was observed in Korean Jindo Black. This results signals increasing inbreeding rate over time.

Artificial breeding, or domestication can cause a reduction in effective population sizes [57,58]. Thus, the observed effect may be due to the number of breeding programs that have been introduced recently, and could be related to the observed heterozygosity reduction. The study conducted by Calboli et al, [59] revealed adverse consequences (loss of unique genetic variants, high prevalence of recessive genetic disorders) of increasing inbreeding rates and a dramatic effect of breeding patterns on genetic diversity based on pedigree information. These results are in accordance with the findings of our study.

It has been noted previously that populations of breeds or species require a minimum effective population size of about 50 or 100 [60]. Therefore, the declining effective population sizes of Korean dogs, especially, the Korean Poongsan White and Korean Donggyeonng White emphasize the need for strong actions and strategies to increase the effective population size while maintaining the genetic diversity these breeds.

## Conclusion

This study presents some interesting findings on the diversity, population structure, ancestral admixture, and demographic history of Korean dog breeds. Since there are few studies on the ancestry and diversity of Korean dog breeds, our study helps to fill gaps in knowledge this population. Korean dogs have clear genetic divergence from modern breeds. The unique genetic structure of Korean dogs has caused them to have extremely distinctive characteristics. It is clear that the effective population size of Korean dogs has decreased from the past to present due to increased inbreeding due to modern breeding programs.

The present results emphasize that Korean dogs have a close relationship with ancient Chinese and Japanese breeds. Since most analyses in the study showed a strong relationship between Korean and Chinese breeds, migration of dogs between China and Korea can be scientifically validated by our study. Therefore, this study suggests Chinese ancestry for Korean dogs. The geographical location, previous studies and the history of these two countries support this hypothesis. Moreover, Korean breeds show a closer relationship with ancient dog breeds than the wolf ancestor. Therefore, we suggest that Korean dogs are also one of the indigenous dog categories that can be considered as the basis of the East-Asian dog domestication process. The various types of admixture events leading to increased diversity of Asian dogs including Korean dogs is greater than in any other part of the world. Korean Donggyeong has a different genetic composition from than other Korean breeds. More studies using whole genome sequencing data, larger sample size and more Korean dog varieties are needed to improve accuracy and to investigate the exact time period for Korean dog domestication.

## Supporting information

S1 Table Dog classification with sample sizes used in this analysis. (DOCX)

S1 Fig Plot of residuals from TreeMix analysis depicted in Fig 5. (TIF)

S2 Fig Inferred dog tree with migration events (three migrations). (TIF)

S3 Fig Inferred dog tree with migration events (five migrations). (TIF)

S4 Fig Inferred dog tree with migration events (seven migrations). (TIF)

S1 File SNP information of Korean breeds (.bed file). (BED)

S2 File SNP information of Korean breeds (.bim file). (BIM)

S3 File SNP information of Korean breeds (.fam file). (FAM)

## References

[1] Serpell J, clutton-Broke J, Coppinger R, Schneider R, Willis MB, Benjamin L, et al The Domestic Dog: Its evolution Behavior and Interactions with people. In Serpell J, editor. Cambridge University Press, Camebridge;1995 p.7–20.

[2] Wayne RK. Molecular evolution of the dog family. Trends in genetics: TIG. 1993;9(6):218–24. Epub 1993/06/01. . 8337763 10.1016/0168-9525(93)90122-x

[3] Vilà C, Savolainen P, Maldonado JE, Amorim IR, Rice JE, Honeycutt RL, et al Multiple and Ancient Origins of the Domestic Dog. Science. 1997;276(5319):1687–9. doi: 10.1126/science.276.5319.1687 9180076 10.1126/science.276.5319.1687

[4] Lindblad-Toh K, Wade CM, Mikkelsen TS, Karlsson EK, Jaffe DB, Kamal M, et al Genome sequence, comparative analysis and haplotype structure of the domestic dog. Nature. 2005;438(7069):803–19. doi: 10.1038/nature04338 16341006 10.1038/nature04338

[5] Shannon LM, Boyko RH, Castelhano M, Corey E, Hayward JJ, McLean C, et al Genetic structure in village dogs reveals a Central Asian domestication origin. Proceedings of the National Academy of Sciences. 2015;112(44):13639–44. doi: 10.1073/pnas.1516215112 26483491 10.1073/pnas.1516215112 PMC4640804

[6] Savolainen P, Zhang Y-p, Luo J, Lundeberg J, Leitner T. Genetic Evidence for an East Asian Origin of Domestic Dogs. Science. 2002;298(5598):1610–3. doi: 10.1126/science.1073906 12446907 10.1126/science.1073906

[7] Frantz LAF, Mullin VE, Pionnier-Capitan M, Lebrasseur O, Ollivier M, Perri A, et al Genomic and archaeological evidence suggest a dual origin of domestic dogs. Science. 2016;352(6290):1228–31. doi: 10.1126/science.aaf3161 27257259 10.1126/science.aaf3161

[8] Parker HG, Kim LV, Sutter NB, Carlson S, Lorentzen TD, Malek TB, et al Genetic Structure of the Purebred Domestic Dog. Science. 2004;304(5674):1160–4. doi: 10.1126/science.1097406 15155949 10.1126/science.1097406

[9] Larson G, Karlsson EK, Perri A, Webster MT, Ho SYW, Peters J, et al Rethinking dog domestication by integrating genetics, archeology, and biogeography. Proceedings of the National Academy of Sciences. 2012;109(23):8878–83. doi: 10.1073/pnas.1203005109 22615366 10.1073/pnas.1203005109 PMC3384140

[10] Cho GJ. Microsatellite Polymorphism and Genetic Relationship in Dog Breeds in Korea. Asian-Australas J Anim Sci. 2005;18(8):1071–4. doi: 10.5713/ajas.2005.1071

[11] Kim KS, Tanabe Y, Park CK, Ha JH. Genetic variability in East Asian dogs using microsatellite loci analysis. The Journal of heredity. 2001;92(5):398–403. Epub 2002/01/05. . 11773246 10.1093/jhered/92.5.398

[12] HA JH. and Kim KS. A review on the origin of Korean native dogs. Korean Journal of Animal Science.1998 40p 701–710

[13] Ha JH, Lee SE, Tak YB and Kim JB. The physical characteristics and blood protein of Korean native dogs. Korean journal of animal science.1998 40; 711–720

[14] Lee CG, Lee JI, Lee CY, Sun SS. A Review of the Jindo, Korean Native Dog—Review. Asian-Australas J Anim Sci. 2000;13(3):381–9. doi: 10.5713/ajas.2000.381

[15] Choi SG, Sung GC, Lee EW, Park ST, Cho GJ, and Song HB. Historical origin on Korean native donggyeong-I dogs. Korean Journal of Companion animal science.2008 5;7–77

[16] Lee E-W, Choi S-K, Cho G-J. Molecular Genetic Diversity of the Gyeongju Donggyeong Dog in Korea. The Journal of Veterinary Medical Science. 2014;76(10):1359–65. doi: 10.1292/jvms.14-0189. PMC4221169. 25030603 10.1292/jvms.14-0189 PMC4221169

[17] Akey JM, Ruhe AL, Akey DT, Wong AK, Connelly CF, Madeoy J, et al Tracking footprints of artificial selection in the dog genome. Proceedings of the National Academy of Sciences. 2010;107(3):1160–5. doi: 10.1073/pnas.0909918107 20080661 10.1073/pnas.0909918107 PMC2824266

[18] Salmon Hillbertz NHC, Isaksson M, Karlsson EK, Hellmen E, Pielberg GR, Savolainen P, et al Duplication of FGF3, FGF4, FGF19 and ORAOV1 causes hair ridge and predisposition to dermoid sinus in Ridgeback dogs. Nat Genet. 2007;39(11):1318–20. http://www.nature.com/ng/journal/v39/n11/suppinfo/ng.2007.4_S1.html. doi: 10.1038/ng.2007.4 17906623 10.1038/ng.2007.4

[19] Ding ZL, Oskarsson M, Ardalan A, Angleby H, Dahlgren LG, Tepeli C, et al Origins of domestic dog in Southern East Asia is supported by analysis of Y-chromosome DNA. Heredity. 2012;108(5):507–14. http://www.nature.com/hdy/journal/v108/n5/suppinfo/hdy2011114s1.html. doi: 10.1038/hdy.2011.114 22108628 10.1038/hdy.2011.114 PMC3330686

[20] vonHoldt BM, Pollinger JP, Lohmueller KE, Han E, Parker HG, Quignon P, et al Genome-wide SNP and haplotype analyses reveal a rich history underlying dog domestication. Nature. 2010;464(7290):898–902. doi: 10.1038/nature08837. PMC3494089. 20237475 10.1038/nature08837 PMC3494089

[21] Maniatis T, Fritsch EF, Sambrook J. Molecular cloning: a laboratory manual: Cold Spring harbor laboratory Cold Spring Harbor, NY; 1982.

[22] Chang C, Chow C, Tellier L, Vattikuti S, Purcell S, Lee J. Software and Supporting Material for "Second-generation PLINK: Rising to the Challenge of Larger and Richer Datasets". GigaScience Database. 2015 4(1):7 http://dx.doi.org/10.5524/100116. 10.1186/s13742-015-0047-8 PMC4342193 25722852

[23] Weir BS, Cockerham CC. Estimating F-statistics for the analysis of population structure. Evolution. 1984;38 doi: 10.2307/2408641 10.1111/j.1558-5646.1984.tb05657.x 28563791

[24] Nei M. Estimation of Average Heterozygosity and Genetic Distance from a Small Number of Individuals. Genetics. 1978;89(3):583–90. PMC121385 17248844 10.1093/genetics/89.3.583 PMC1213855

[25] Goudet J. hierfstat, a package for r to compute and test hierarchical F-statistics. Molecular Ecology Notes. 2005;5(1):184–6. doi: 10.1111/j.1471-8286.2004.00828. x

[26] Reich D, Thangaraj K, Patterson N, Price AL, Singh L. Reconstructing Indian population history. Nature. 2009;461(7263):489–94. http://www.nature.com/nature/journal/v461/n7263/suppinfo/nature08365_S1.htm doi: 10.1038/nature08365 19779445 10.1038/nature08365 PMC2842210

[27] Yang J, Lee S, Goddard M, Visscher P. Gcta: A tool for genome-wide complex trait analysis. Am J Hum Genet. 2011;88 doi: 10.1016/j.ajhg.2010.11.011 21167468 10.1016/j.ajhg.2010.11.011 PMC3014363

[28] Team RC. R: A language and environment for statistical computing. R Foundation for Statistical Computing, Vienna, Austria 2013. 2014.

[29] Alexander DH, Novembre J, Lange K. Fast model-based estimation of ancestry in unrelated individuals. Genome Research. 2009;19(9):1655–64. doi: 10.1101/gr.094052.109. PMC2752134. 19648217 10.1101/gr.094052.109 PMC2752134

[30] Lee T-H, Guo H, Wang X, Kim C, Paterson AH. SNPhylo: a pipeline to construct a phylogenetic tree from huge SNP data. BMC Genomics. 2014;15(1):162 doi: 10.1186/1471-2164-15-162 24571581 10.1186/1471-2164-15-162 PMC3945939

[31] http://tree.bio.ed.ac.uk/software/figtree/.

[32] Pickrell JK, Pritchard JK (2012) Inference of Population Splits and Mixtures from Genome-Wide Allele Frequency Data. PLoS Genet 8(11): e1002967 doi: 10.1371/journal.pgen.1002967 23166502 10.1371/journal.pgen.1002967 PMC3499260

[33] 26. Reich D, Thangaraj K, Patterson N, Price AL, Singh L. Reconstructing Indian population history. Nature. 2009;461(7263):489–94. http://www.nature.com/nature/journal/v461/n7263/suppinfo/nature08365_S1.htm doi: 10.1038/nature08365 19779445 10.1038/nature08365 PMC2842210

[34] Sved JA. Linkage disequilibrium and homozygosity of chromosome segments in finite populations. Theoretical Population Biology. 1971;2(2):125–41. http://dx.doi.org/10.1016/0040-5809(71)90011-6. 5170716 10.1016/0040-5809(71)90011-6

[35] Hill WG, Robertson A. Linkage disequilibrium in finite populations. Theoretical and Applied Genetics. 1968;38(6):226–31. doi: 10.1007/BF01245622 24442307 10.1007/BF01245622

[36] Barbato M, Orozco-terWengel P, Tapio M and Bruford MW (2015). SNeP: a tool to estimate trends in recent effective population size trajectories using genome-wide SNP data. Front. Genet. 6:109 doi: 10.3389/fgene.2015.00109 25852748 10.3389/fgene.2015.00109 PMC4367434

[37] Boyko AR. The domestic dog: man's best friend in the genomic era. Genome Biology. 2011;12(2):216 doi: 10.1186/gb-2011-12-2-216 21338479 10.1186/gb-2011-12-2-216 PMC3188790

[38] Vaysse A, Ratnakumar A, Derrien T, Axelsson E, Rosengren Pielberg G, Sigurdsson S, et al (2011) Identification of Genomic Regions Associated with Phenotypic Variation between Dog Breeds using Selection Mapping. PLoS Genet 7(10): e1002316 doi: 10.1371/journal.pgen.1002316 22022279 10.1371/journal.pgen.1002316 PMC3192833

[39] Lachance J, Tishkoff SA. SNP ascertainment bias in population genetic analyses: why it is important, and how to correct it. BioEssays: news and reviews in molecular, cellular and developmental biology. 2013;35(9):780–6. Epub 2013/07/10. doi: 10.1002/bies.201300014 ; PubMed Central PMCID: PMCPMC3849385. 23836388 10.1002/bies.201300014 PMC3849385

[40] Gravel S, Henn BM, Gutenkunst RN, Indap AR, Marth GT, Clark AG, et al Demographic history and rare allele sharing among human populations. Proc Natl Acad Sci U S A. 2011;108(29):11983–8. Epub 2011/07/07. doi: 10.1073/pnas.1019276108 ; PubMed Central PMCID: PMCPMC3142009. 21730125 10.1073/pnas.1019276108 PMC3142009

[41] Novembre J, Johnson T, Bryc K, Kutalik Z, Boyko AR, Auton A, et al Genes mirror geography within Europe. Nature. 2008;456(7218):98–101. doi:http://www.nature.com/nature/journal/v456/n7218/suppinfo/nature07331_S1.html. doi: 10.1038/nature07331 18758442 10.1038/nature07331 PMC2735096

[42] Lao O, Lu TT, Nothnagel M, Junge O, Freitag-Wolf S, Caliebe A, et al Correlation between genetic and geographic structure in Europe. Current biology: CB. 2008;18(16):1241–8. Epub 2008/08/12. doi: 10.1016/j.cub.2008.07.049 . 18691889 10.1016/j.cub.2008.07.049

[43] Lee E-W, Choi S-K, Cho G-J. Molecular Genetic Diversity of the Gyeongju Donggyeong Dog in Korea. The Journal of Veterinary Medical Science. 2014;76(10):1359–65. doi: 10.1292/jvms.14-0189. PMC4221169. 25030603 10.1292/jvms.14-0189 PMC4221169

[44] 23. Lee S-H, Park B-H, Sharma A, Dang C-G, Lee S-S, Choi T-J, et al Hanwoo cattle: origin, domestication, breeding strategies and genomic selection. Journal of Animal Science and Technology. 2014;56(1):2 doi: 10.1186/2055-0391-56-2 26290691 10.1186/2055-0391-56-2 PMC4534185

[45] Wang G-D, Zhai W, Yang H-C, Wang L, Zhong L, Liu Y-H, et al Out of southern East Asia: the natural history of domestic dogs across the world. Cell Res. 2016;26(1):21–33. doi: 10.1038/cr.2015.147 26667385 10.1038/cr.2015.147 PMC4816135

[46] Yang H, Wang G, Wang M, Ma Y, Yin T, Fan R, et al The origin of chow chows in the light of the East Asian breeds. BMC Genomics. 2017;18(1):174 doi: 10.1186/s12864-017-3525-9 28201986 10.1186/s12864-017-3525-9 PMC5312535

[47] Wang L, Ma Y-P, Zhou Q-J, Zhang Y-P, Savolainen P, Wang G-D. The geographical distribution of grey wolves (Canis lupus) in China: a systematic review. Zoological Research. 2016;37(6):315–26. doi: 10.13918/j.issn.2095-8137.2016.6.315. PMC5359319. 28105796 10.13918/j.issn.2095-8137.2016.6.315 PMC5359319

[48] Fan Z, Silva P, Gronau I, Wang S, Armero AS, Schweizer RM, et al Worldwide patterns of genomic variation and admixture in gray wolves. Genome Research. 2016;26(2):163–73. doi: 10.1101/gr.197517.115 26680994 10.1101/gr.197517.115 PMC4728369

[49] Tanabe Y. The Origin of Japanese Dogs and their Association with Japanese People. Zoological Science, 8, 639–651. (1991). Retrieved from http://biostor.org/reference/106761.;

[50] Freedman AH, Gronau I, Schweizer RM, Ortega-Del Vecchyo D, Han E, Silva PM, et al (2014) Genome Sequencing Highlights the Dynamic Early History of Dogs. PLoS Genet 10(1): e1004016 doi: 10.1371/journal.pgen.1004016 24453982 10.1371/journal.pgen.1004016 PMC3894170

[51] Gutierrez JP, Cervantes I, Molina A, Valera M, Goyache F. Individual increase in inbreeding allows estimating effective sizes from pedigrees. Genet Sel Evol. 2008;40(4):359–78. Epub 2008/06/19. doi: 10.1051/gse:2008008 ; PubMed Central PMCID: PMCPMC2674907. 18558071 10.1186/1297-9686-40-4-359 PMC2674907

[52] Crow JF, Kimura M. An introduction to population genetics theory. (1970) Burgess Publishing,Minneapolis

[53] Falconer DS, Mackay FC. Introduction to Quantitative Genetics, 4^th^ ed., (1996).Longman Group Ltd, England.

[54] FAO. (1996). Secondary guidelines for development of national farm animal genetic resources management plans: Management of small populations at risk. UN Food and Agric. Org https://www.google.co.kr/url?sa=t&rct=j&q=&esrc=s&source=web&cd=1&cad=rja&uact=8&ved=0ahUKEwiNp4K1s5DXAhVIurwKHT3dB8UQFggmMAA&url=http%3A%2F%2Fwww.fao.org%2F3%2Fa-w9361e.pdf&usg=AOvVaw2hNviO56VxwVz-lWBr_ucZ

[55] Schwartz MK, Tallmon DA, Luikart G. Review of DNA-based census and effective population size estimators. Animal Conservation. 1998;1(4):293–9. doi: 10.1111/j.1469-1795. 1998.tb00040. x

[56] Alam M, Han KI, Lee DH, Ha JH, Kim JJ. Estimation of Effective Population Size in the Sapsaree: A Korean Native Dog (Canis familiaris). Asian-Australas J Anim Sci. 2012;25(8):1063–72. doi: 10.5713/ajas.2012.12048 25049664 10.5713/ajas.2012.12048 PMC4093000

[57] Nomura T, Honda T, Mukai F. Inbreeding and effective population size of Japanese Black cattle. J Anim Sci. 2001;79(2):366–70. Epub 2001/02/24. . 11219445 10.2527/2001.792366x

[58] Uimari P, Tapio M. Extent of linkage disequilibrium and effective population size in Finnish Landrace and Finnish Yorkshire pig breeds. J Anim Sci. 2011;89(3):609–14. Epub 2010/11/03. doi: 10.2527/jas.2010-3249 . 21036932 10.2527/jas.2010-3249

[59] Calboli FCF, Sampson J, Fretwell N, Balding DJ. Population Structure and Inbreeding From Pedigree Analysis of Purebred Dogs. Genetics. 2008;179(1):593–601. doi: 10.1534/genetics.107.084954. PMC2390636. 18493074 10.1534/genetics.107.084954 PMC2390636

[60] Meuwissen T. Genetic management of small populations: A review. Acta Agriculturae Scandinavica, Section A—Animal Science. 2009;59(2):71–9. doi: 10.1080/09064700903118148 10.1080/09064700902988905 PMC4936439 27453634

