# Supplementary material for: Genome-wide analysis of the diversity and ancestry of Korean dogs
Source: PLoS One. 2017 Nov 28;12(11):e0188676. doi: 10.1371/journal.pone.0188676 (PMC5705110; doi:10.1371/journal.pone.0188676)
Supplement: S1 Table — (DOCX) [file pone.0188676.s001.docx]

**S1 Table. Dog classification with samples size used in the analysis.**

| Breed | Abbreviation | No. of animals |
| --- | --- | --- |
| ***Non-dogs*** | | |
| Coyote | CFC | 7(a) |
| Gray wolf | GRW | 14(a) |
| Korean wolf | KRW | 3(a) |
| Chines wolf | CHW | 9(a) |
| European wolf | EUW | 22(a) |
| Mediterranean wolf | MEW | 9(a) |
| Russian wolf | RUW | 13(a) |
| US wolf | USW | 11(a) |
| ***Korean breeds*** | | |
| Korean Donggyengi White (Dong) | KDW | 52 |
| Korean Jindo Brindle | KJD | 11 |
| Korean Jindo Black | KJB | 32 |
| Korean Jindo White | KJW | 42 |
| Korean Jindo Black and Tan | KJT | 32 |
| Korean Poongsan White | KPW | 19 |
| ***Ancient breeds*** | | |
| Akita | AKT | 12(a) |
| Chow Chow | CHO | 6(a) |
| Chines Shar Pei | CHS | 8(a) |
| Lhasa Apso | LHA | 15(a) |
| Basenji | BSJ | 30(a) |
| Afghan Hound | AFH | 11(a) |
| Alaskan Malamute | ALM | 12(a) |
| Saluki | SAL | 7(a) |
| Pekingese | PEK | 13(a) |
| Shiba Inu | SHI | 8(a) |
| Shih Tzu | SHT | 27(a) |
| Siberian Husky | SIH | 17(a) |
| Tibetan Terrier | TIT | 7(a) |
| ***Modern breeds*** | | |
| Border Collie | BDC | 52(a) |
| Boxer | BOX | 134(a) |
| Cavalier king Charles spaniel | CAV | 52(a) |
| Chinese Crested | CHC | 13(a) |
| Chihuahua | CHH | 14(a) |
| Croatian | CRS | 3(a) |
| English Setter | ENS | 85(a) |
| English Springer spaniel | ESS | 106(a) |
| Great Dane | GRD | 19(a) |
| Golden retriever | GRT | 291(a) |
| German shepherd | GSD | 270(a) |
| Japanese chin | JPC | 4(a) |
| Labrador retriever | LRT | 507(a) |
| Maltese | MAL | 85(a) |
| Miniature pinscher | MNP | 18(a) |
| Miniature schnauzer | MNS | 60(a) |
| Newfoundland | NEF | 79(a) |
| Poodle | POO | 25(a) |

^a^ Shannon et al. 2015
