# Supplementary figures and images for: Genome-wide analysis of the diversity and ancestry of Korean dogs
Source: PLoS One. 2017 Nov 28;12(11):e0188676. doi: 10.1371/journal.pone.0188676 (PMC5705110; doi:10.1371/journal.pone.0188676)

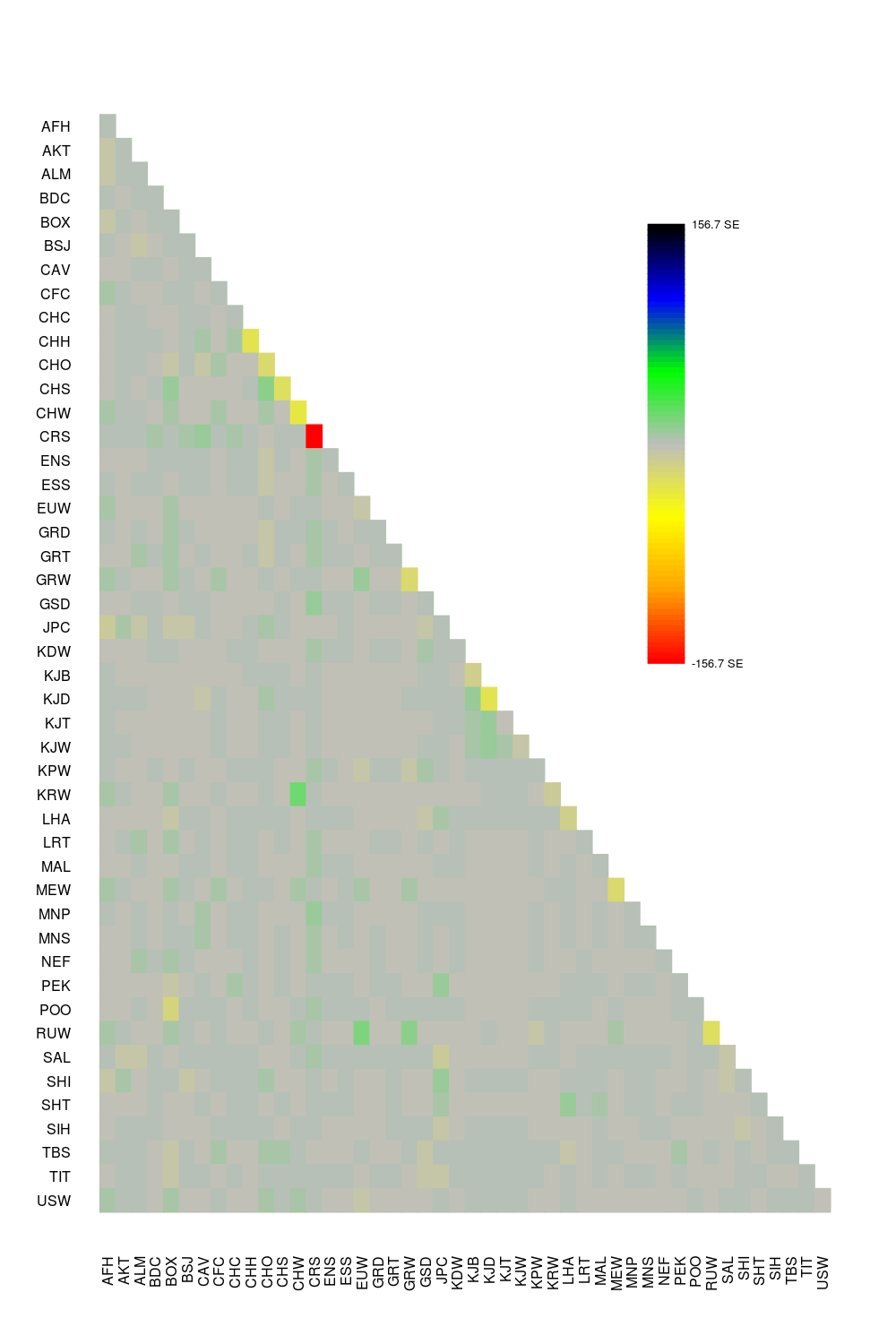

Supplement: S1 Fig — (TIF) [file pone.0188676.s002.tif]

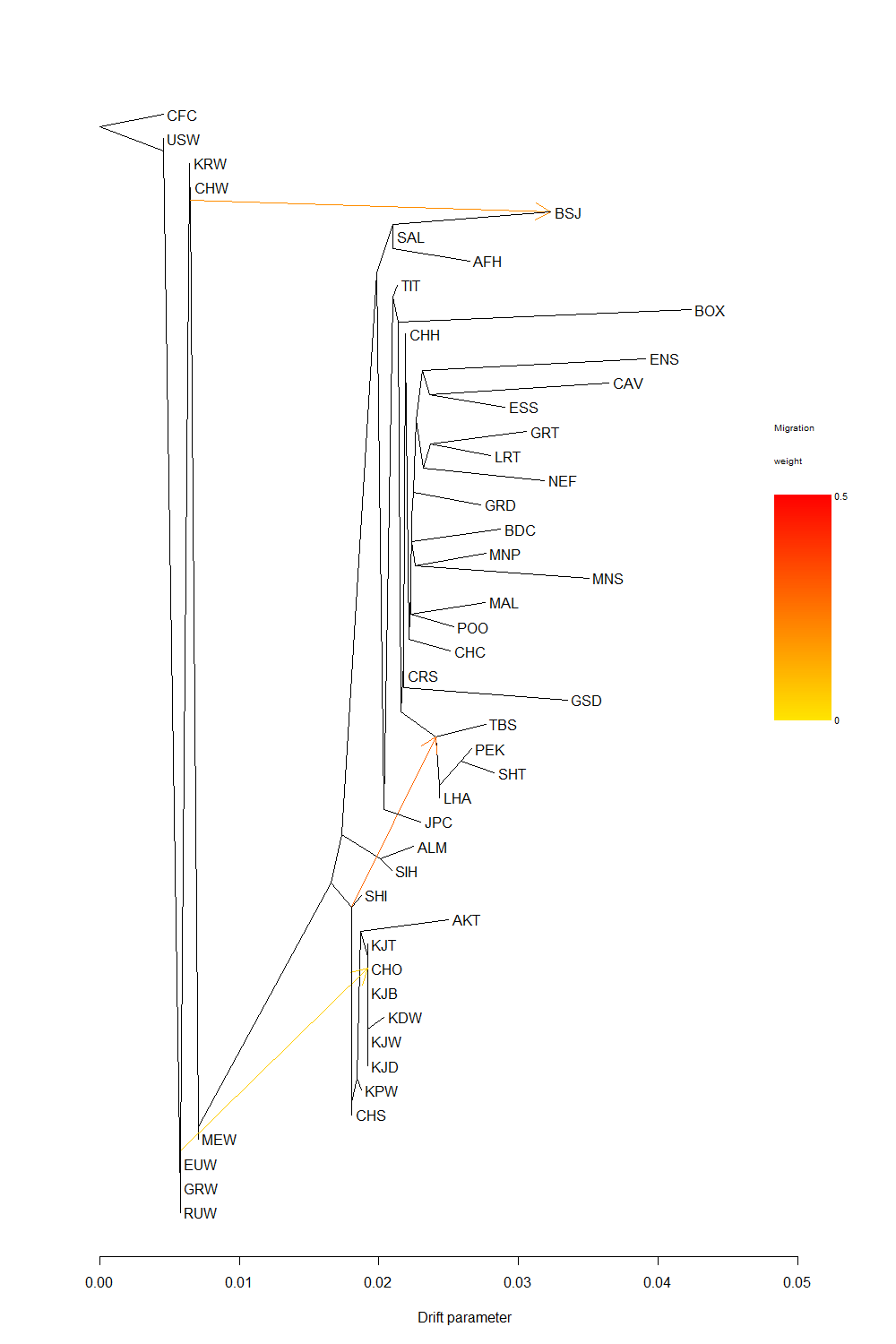

Supplement: S2 Fig — (TIF) [file pone.0188676.s003.tif]

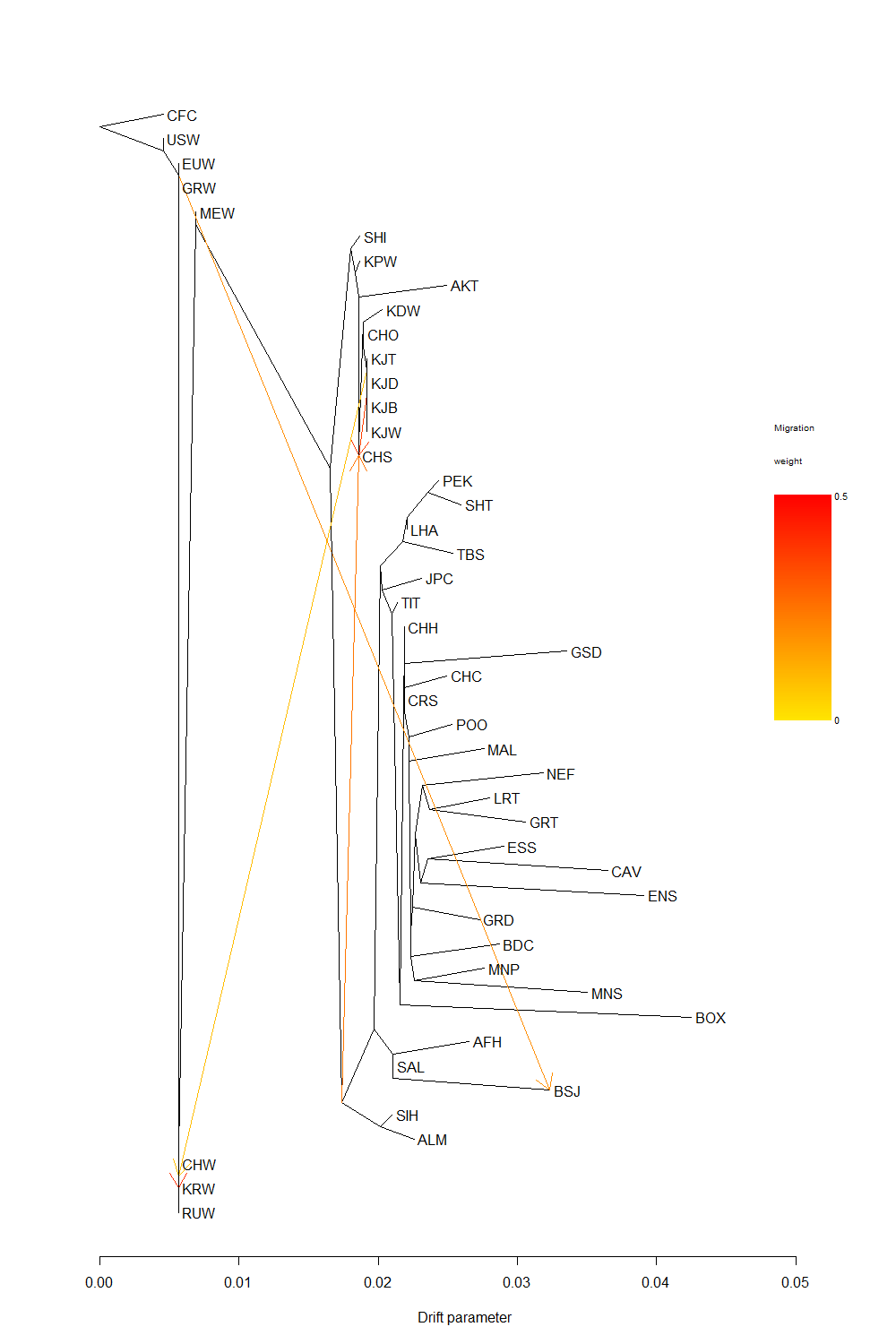

Supplement: S3 Fig — (TIF) [file pone.0188676.s004.tif]

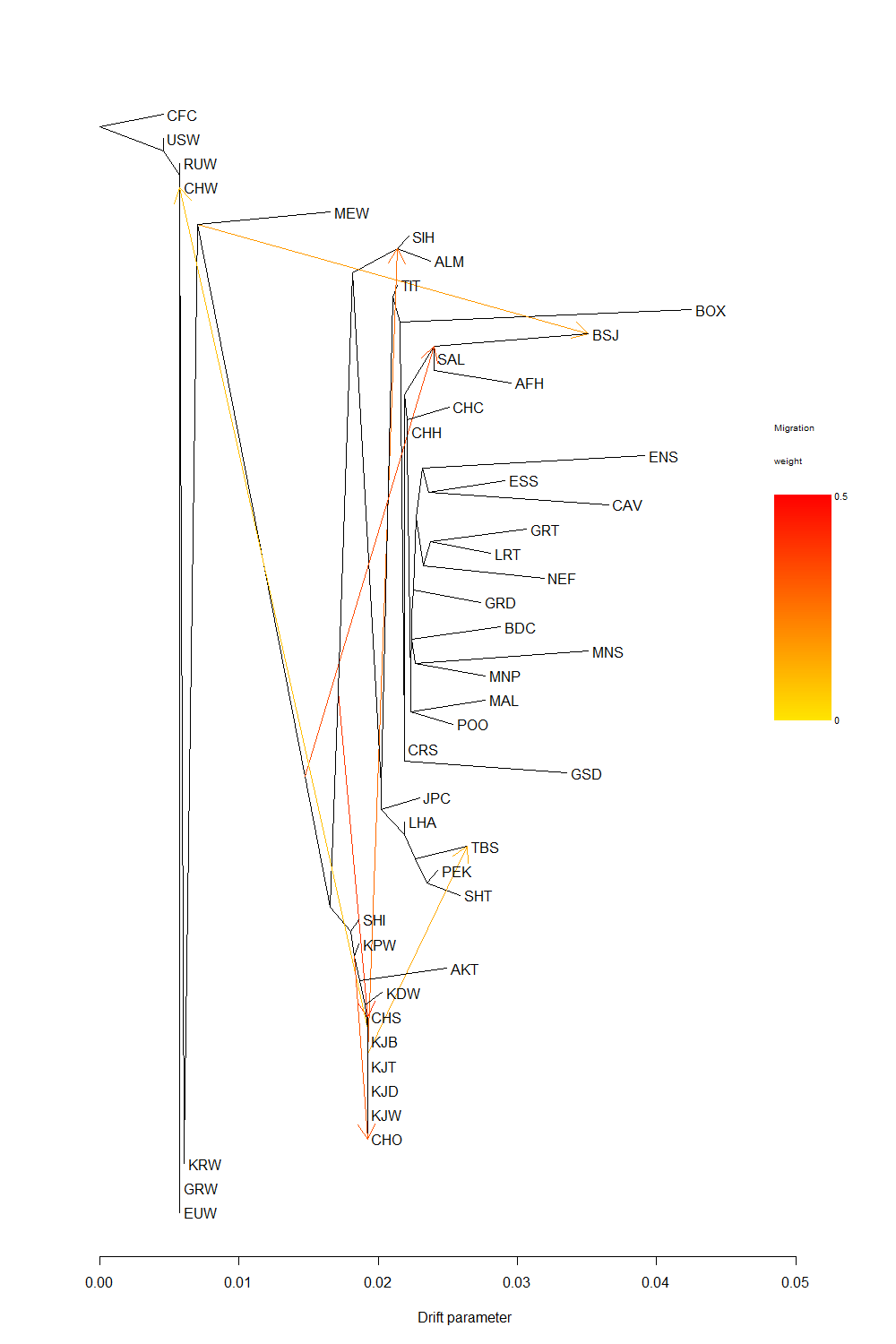

Supplement: S4 Fig — (TIF) [file pone.0188676.s005.tif]
